# Two mutations in the axonemal dynein heavy chain gene 5 in a Chinese asthenozoospermia patient: A case report

**DOI:** 10.1097/MD.0000000000020813

**Published:** 2020-07-10

**Authors:** Jing He, Leilei Li, Yang Yu, Xiaonan Hu, Hongguo Zhang, Ruizhi Liu, Ruixue Wang

**Affiliations:** Center for Reproductive Medicine and Center for Prenatal Diagnosis, First Hospital, Jilin University, Changchun, Jilin, P.R. China.

**Keywords:** asthenospermia, bioinformatics analysis, dynein heavy chain gene5, dyneins, gene

## Abstract

**Introduction:**

As one of the most common causes of male infertility, asthenozoospermia mainly shows low sperm motility, accounting for 81.84% of male infertility patients. Recently, there has been a notable increase for relationship between genetic testing and asthenozoospermia. In this report, we design to provide clues to prove relationship between dynein heavy chain gene 5 (*DNAH5)* gene alterations and asthenozoospermia. This also provides a reference for patients to choose a reasonable treatment plan or genetic counseling to assist reproductive reproduction.

**Patients concern:**

In the present study, we screened 143 patients with asthenozoospermia for variants in *DNAH5* gene. We used high-throughput targeted gene sequencing technology and the data were assessed by bioinformatics analysis.

**Diagnosis:**

We found 1 of 143 asthenozoospermia patients was detected as carrying *DNAH5* compound heterozygous variants (c.3502G>A and c.2578–11_2578-7del).

**Outcomes:**

The variation c.2578-11_2578-7del was predicted in silico to not affect the splicing by HSF3. The variation c.3502G > A (p.E1168K) may cause disease by Mutationtaster software. They may contribute to a risk of male infertility in Chinese patients.

**Conclusions:**

We discussed the possible association between mutations in *DNAH5* and asthenospermia for the first time in Chinese people. If confirmed in larger samples and different races, this result was meaningful for a better diagnosis of asthenospermia patients.

## 1 Introduction

Infertility is the third most serious disease behind the cancer and cardiovascular disease, and it has received worldwide attention. Infertility is caused by male factors and accounts for about half of infertile couples. As one of the most common causes of male infertility, asthenozoospermia mainly shows low sperm motility, accounting for 81.84% of male infertility patients^[1]^; some patients are accompanied by oligozoospermia or teratozoospermia. There are many factors leading to the development of asthenozoospermia, including autoimmune factors, endocrine factors, infectious factors, environmental factors, and genetic factors. After clinical testing and diagnosis, doctors can perform selective drug therapy or assisted reproductive technology for infertile patients, but some asthenozoospermia patients are still difficult to be determined the cause. Conventional genetic testing techniques, such as karyotype analysis, fluorescence in situ hybridization, and gene chip cannot effectively diagnose the cause of genetics, and it is difficult to meet the clinical needs of patients. With the development and clinical application of molecular genetic technology, it is gradually becoming a hot topic to explore the pathogenesis of asthenozoospermia from the perspective of molecular genetics. This also provides a reference for patients to choose a reasonable treatment plan or genetic counseling to assist reproductive reproduction.

For the clinical diagnosis of asthenospermia, sperm motility is an important indicator. Sperm motility mainly depends on the movement of the sperm tail, which is known to be significantly correlated with sperm motility, and sperm can generate forward momentum by swinging through the flagella of the tail. Any abnormal mechanism that affects the composition of the flagellum region will lead to decreasing sperm motility, leading to the occurrence of asthenospermia. According to the OMIM database search results, *DNAH5* gene is located on human chromosome 5p15.2, containing 79 exon sequences, with a total length of about 250Kb. The encoded dynein is involved in the assembly of the outer axon dynein arm of flagella or cilia. Olbrich et al^[2]^ isolated *DNAH5* cDNA fragments from gene libraries of testes and trachea by PCR amplification techniques; and detected *DNAH5* expression in organs such as brain, heart, and testis using Northern acrobatic techniques. 90% of men with primary ciliary dyskinesia (PCD) or Kartagener syndrome accompanied by asthenozoospermia, the majority of them presenting dynein genes mutation.^[3,4]^ To date, Zuccarello et al^[5]^ screened 90 patients with isolated nonsyndromic asthenozoospermia for mutations in *DNAH5* and other genes. They found that the *DNAH5* gene is specifically associated with asthenozoospermia at a frequency of 1.1% (1/90).^[5]^ In this case report, it provide clues to prove a relationship between *DNAH5* gene alterations and asthenozoospermia.

## 2 Materials and methods

### 2.1 Patients

The 143 subjects were treated from the Department of Reproductive Center and Prenatal Diagnosis Center of the First Hospital of Jilin University from May 2011 to December 2016. Two consecutive semen analyses were diagnosed as patients with asthenozoospermia. After initial screening, the conditions were as follows: no severe varicocele; no infection, genital trauma, major illness, and other past medical history; no long-term radiation exposure or radiotherapy and chemotherapy treatment; chromosome karyotype no abnormality; no AZF microdeletion in chromosomes Y; no hyperthermia in the diagnosis period. The study was approved by the Ethics Committee of the First Hospital of Jilin University (2017–399). Patient has provided informed consent for publication of the case.

### 2.2 *DNAH5* gene sequencing

Genomic DNA extraction in blood was used by BloodGen Midi kits (Kangwei Century Biological Technology Co., Ltd., Beijing, PR China). Genomic DNA sequencing was carried out using exome capture using the in house Targeted genes Panel (Peking Medriv Academy of Genetics and Reproduction, Peking) followed by next-generation sequencing on the Illumina MiSeq sequencing platform(Illumina, San Diego, CA). According to references and the OMIM database (http://www.omim.org), we established the *DNAH5* probes by asthenozoospermia-associated genes of published reports. We used Cutadapt (https://pypi.python.org/pypi/cutadapt) and FastQC (https://www.bioinformatics.babraham.ac.uk/projects/fastqc/) excluding the small low-quality fragments and a 30/50 linker. Then preprocessed clean reads were compared with the hg19 human reference sequence using BWA software (http://bio-bwa.source forge.net). Duplicated reads from library and PCR preparation were removed with Picard tools.

The single-nucleotide variants (SNVs) and Indel variants in the preprocessed sequence information were found using the Genome Analysis Tool Kit (GATK) program (https://www.broad institute.org/gatk). To ensure that the sequencing results are high-quality data, the output data were evaluated, and the data to be detected needed to meet the following indicators: 100% ratio >95%; 100% repetition rate <20%; coverage 20× reading depth was 92% to 99.99%; the average coverage of the target area was >80×. Functional annotations and frequent annotations were performed using the ANNOVAR program (annovar.openbioinformatics.org/en/latest/). The former included gene regions, variation effects, amino acid changes, location on chromosomes, among others; the frequent annotations were filtered using the 1000 Genomes (http://www.1000genomes.org/data), Exome Variation Server (http://evs.gs.washington.edu/EVS/), Exome Aggregation Consortium (http://exac.broadinstitute.org/), and dbSNP databases (http://www.ncbi.nlm.nih.gov/snp). In addition to synonymous variations, both rare and novel variations were reviewed for further investigation. For the analysis of SNVs, SIFT (https://sift.jcvi.org/), PolyPhen-2 (http://genetics.bwh.harvard.edu/pph2/), and Mutation Taster2 (http://www.mutationtaster.org) algorithms were used to predict the no synonymous variations that would damage protein function. Mutation Taster2 was also used to assess frame shift variation and the harmfulness to splicing of mutations close to splice sites was also predicted using Human Splicing Finder 3.1 (http://www.umd.be/HSF3/). The results were further validated with the use of Sanger sequencing (BGI, Shenzhen, China)

## 3 Results

### 3.1 Basic clinical information

A total of 143 patients with asthenozoospermia were included in the study. All patients were of primary infertility, and there were no abnormalities in karyotype results and AZF results. One patient found a potentially pathogenic SNV in the *DNAH5* gene, which was 0.70% (1/143). The patient was 26, coming to our center for medical treatment due to 2 infertility years. His peripheral blood serum reproductive hormone results were follicle-stimulating hormone (FSH) 14.1 mIU/mL, luteinizing hormone 8.4mIU/mL, estradiol 2 39.17 pg/mL, prolactin 250 μIU/mL, testosterone 14nmol/L. In addition, his conventional semen analysis showed that sperm concentration was 3.30 × 10^6^ cells/mL, sperm vitality was 26.53%. He was diagnosed as having oligoaasthenozoospermia and other indicators are shown in Table 1.

**Table 1:**
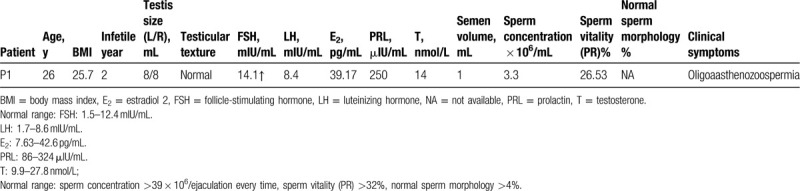
General clinical information and peripheral blood serum reproductive hormone test results and conventional semen analysis.

### 3.2 *DNAH5* gene mutation result

**Figure 1:**
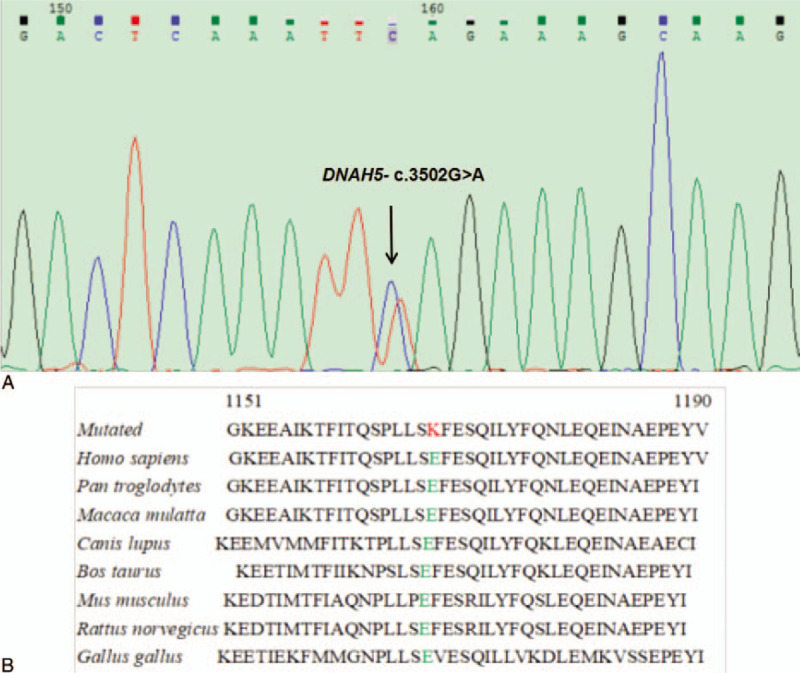
(A) *DNAH5* c.3502G > A mutation in patient was confirmed by Sanger sequencing. The position is indicated by an arrow. (B) Multiple sequence alignment in *DNAH5* from different species.

**Figure 2:**
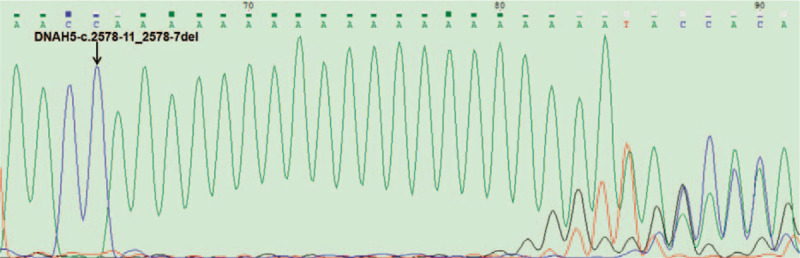
*DNAH5* c.2578-11_2578-7del mutation in patient was confirmed by Sanger sequencing. The position is indicated by an arrow.

This study found that 1 patient carried 2 pathogenic SNVs with the *DNAH5* gene, as shown in Table 2. The mutation c.3502G>A occurs at the 13871769 base position of the p15.2 region of the chromosome 5, resulting in the substitution of the 1168th glutamic acid subunit of the *DNAH5* protein by the lysine subunit. The mutation c.3502G > A was not reported in the dbSNP database and the ExAC database. It was not pathogenic by SIFT and Polyphen-2 software; however, the region of the mutation c.3502G > A was highly conserved among different 8 species as shown in Figure 1B; in addition, Mutationtaster software predicts that it may cause disease. The variation c.2578-11_2578-7del occurs at the 13886245_13886249 base site of the p15.2 region of the chromosome 5, and is located at the 3’ end of the 17th intron region of the *DNAH5* gene, adjacent to the 5’ end of the 18th exon. The variation c.2578-11_2578-7del has been reported in the dbSNP database and ExAC database, and its frequency of occurrence in the population is about 0.01158; it is predicted by HSF3 software that the SNV site is located in the intron region, not affecting the splicing of post-transcriptional mRNA of the DNAH5 gene. Using Sanger sequencing, all variant sites were detected and identified as heterozygous genotypes, consistent with the results of gene capture sequencing; the results of the detected mutations are shown in Figure 1A and Figure 2.

**Table 2:**
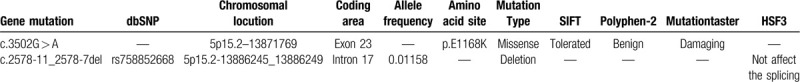
Bioinformatics analysis of *DNAH5* gene mutations by whole-genome sequencing.

**Figure 3:**
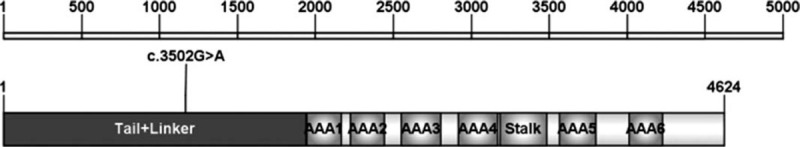
Primary structure model of DNAH5 protein.

Based on data from the NCBI on DNAH5 protein, we created a primary structural model of the DNAH5 protein and identified the location of the variant site. The DNAH5 protein also contains 6 AAA domains (AAA1-6), a coaled-coil stalk domain and a Linker domain, which form a dynamic unit, as shown in Figure 3. The DNAH5 protein consists of a total of 4624 amino acid subunits. In this study, the asthenozoospermia patient who carried DNAH5 compound heterozygous variants (c.3502G > A and c.2578-11_2578-7del) could use assisted reproductive technology to obtain offspring through genetic counseling.

## 4 Discussion

Infertility is defined as the phenomenon that a woman of a suitable age is not able to conceive normally after a period of >1 year of regular sexual life without the use of any contraceptive measures after marriage.^[6]^ About 10% to 15% of married couples around the world are affected by infertility.^[5]^ With the increase of environmental pollution and food safety, the incidence of infertility is on the rise. At present, the number of infertility patients in China has exceeded 40 million; male infertility factors account for about 50%. Most male infertility patients are accompanied by abnormal semen parameters, including: spermatogenesis or no-ejection sperm, low sperm concentration, insufficient sperm motility, and abnormal sperm morphology.^[7]^ According to different abnormal semen parameters, the World Health Organization classifies infertile patients, mainly azoospermia, oligozoospermia, weak sperm disease, and deformed sperm disease.^[8]^

Asthenospermia is a common cause of male infertility and the source of most cases is unknown. Asthenospermia has been found in 90% of PCD male patients, and lots of them showed dynein genes variants.^[3,9]^ This is a report assessing the presence of *DNAH5* gene mutations in patients affected by solitary spermatogenesis. In addition to the normal variants detected in patients and controls, we found 2 mutations specific for asthenospermia (in the *DNAH5* gene) with a frequency of 7.5 ‰ (1/134). All of these mutations result in strongly conserved amino acid substitutions in elite species, indicating their important role in protein structure. In particular, the DNAH5 protein is located in the axon dynein cluster (ODA) of the axon, permanently attached to the A tubule of each external microtubule bimodal and transiently attached to the B tubule of the bimodal peak of the adjacent microtubule to generate slip motion.

In our study, 1 of 143 asthenozoospermia patients was detected as carrying DNAH5 compound heterozygous variants (c.3502G > A and c.2578-11_2578-7del). The variation c.2578-11_2578-7del was predicted in silico to not affect the splicing by HSF3. The variation c.3502G > A (p.E1168K) may cause disease by Mutationtaster software, although it was benign by SIFT and Polyphen-2 software. Heavy chain 5 consists of an *N*-terminal domain that interacts with other intermediate and light chains, a motor domain (core) of the heptameric AAA subdomain with ATP-enzyme function, a linking stalk, and a microtubule-binding domain (mutations E1168K is located in linking stalk). In 2008, Zuccarello et al^[5]^ reported that heterozygous mutations in *DNAH5*, *DNAH11*, and other genes lead to the occurrence of idiopathic asthenozoospermia, but all patients did not show clinical symptoms of PCD, speculating that asthenozoospermia may be PCD syndrome another mild phenotype. In addition, studies have shown that mutations in the DNAH5 gene may cause infertility symptoms such as azoospermia or oligozoospermia.^[4,10]^ At present, the relationship between *DNAH5*, *DNAH11* genes, and male infertility is not clear, but DNAH5 and DNAH11 are important components of the flagellar external axis silk power arm, and are highly conserved among different species; *DNAH5* or *DNAH11* gene mutations will lead to low sperm motility. Pereira et al^[11]^ clinically reported that heterozygous mutations in the *DNAH5* gene lead to defects in sperm motility. The above research supports the association between *DNAH5*, *DNAH11* genes, and asthenozoospermia. And Hornef et al^[12]^ reported that multiple new heterozygous mutation sites on DNAH5 gene including 2 mutations were related to the occurrence of PCD. This patient showed clinical symptom was oligoaasthenozoospermia, and his FSH was 14.1 mIU/mL. Therefore, we hypothesized that the compound heterozygous mutations in patients (c.3502G > A and c.2578-11_2578-7del) strongly influenced the protein function of patients and were associated with the etiology of low sperm motility.

In conclusion, the limitation of the present study is that we did not perform Transmission Electron Microscope studies and could not assess the ultrastructure of sperm flagella. However, we report the possible association between variants in *DNAH5* and asthenospermia for the first time in Chinese people. For the present research, results have showed that if confirmed in larger samples and different races, this result was meaningful for a better diagnosis of asthenospermia patients. Furthermore, many asthenospermia patients are chosen in assisted reproductive technology (Intra Cytoplasmatic Sperm Injection, ICSI), and thus may transmit the disease to their offsprings; so, the asthenospermia patients need genetic counseling in time.

## Acknowledgments

The authors are sincerely grateful to all subjects who participated in this study.

## Author contributions

**Conceptualization:** Ruixue Wang.

**Data curation:** Ruixue Wang.

**Formal analysis:** Hongguo Zhang.

**Investigation:** Yang Yu.

**Methodology:** Xiaonan Hu.

**Project administration:** Ruizhi Liu.

**Writing – original draft:** Jing He.

**Writing – review & editing:** Leilei Li.
